# Uptake and Outcomes of Neoadjuvant Chemotherapy Among US Patients With Less Common Epithelial Ovarian Carcinomas

**DOI:** 10.1001/jamanetworkopen.2023.18602

**Published:** 2023-06-16

**Authors:** Koji Matsuo, Shinya Matsuzaki, Michihide Maeda, Alesandra R. Rau, Kosuke Yoshihara, Ryo Tamura, Muneaki Shimada, Hiroko Machida, Mikio Mikami, Maximilian Klar, Lynda D. Roman, Jason D. Wright, Anil K. Sood, David M. Gershenson

**Affiliations:** 1 Division of Gynecologic Oncology, Department of Obstetrics and Gynecology, University of Southern California, Los Angeles; 2 Norris Comprehensive Cancer Center, University of Southern California, Los Angeles; 3 Department of Gynecology, Osaka International Cancer Institute, Osaka, Japan; 4 Department of Obstetrics and Gynecology, Niigata University School of Medicine, Niigata, Japan; 5 Department of Obstetrics and Gynecology, Tohoku University School of Medicine, Miyagi, Japan; 6 Department of Obstetrics and Gynecology, Tokai University School of Medicine, Kanagawa, Japan; 7 Department of Obstetrics and Gynecology, University of Freiburg Faculty of Medicine, Freiburg, Germany; 8 Division of Gynecologic Oncology, Department of Obstetrics and Gynecology, Columbia University College of Physicians and Surgeons, New York, New York; 9 Department of Gynecologic Oncology and Reproductive Medicine, The University of Texas MD Anderson Cancer Center, Houston

## Abstract

**Importance:**

Randomized clinical trials examining the effectiveness of neoadjuvant chemotherapy (NACT) for advanced ovarian cancer predominantly included patients with high-grade serous carcinomas. The use and outcomes of NACT in less common epithelial carcinomas are understudied.

**Objective:**

To investigate the uptake and survival outcomes in treatment with NACT for less common histologic subtypes of epithelial ovarian cancer.

**Design, Setting, and Participants:**

A retrospective cohort study and systematic literature review with meta-analysis was conducted using the National Cancer Database from 2006 to 2017 and the National Cancer Institute’s Surveillance, Epidemiology, and End Results Program from 2006 to 2019. Data analysis was performed from July 2022 to April 2023. The evaluation included patients with stage III to IV ovarian cancer with clear cell, mucinous, or low-grade serous histologic subtypes who received multimodal treatment with surgery and chemotherapy.

**Exposures:**

Exposure assignment per the sequence of treatment: primary debulking surgery (PDS) followed by chemotherapy (PDS group) or NACT followed by interval surgery (NACT group).

**Main Outcomes and Measures:**

Temporal trends and characteristics of NACT use were assessed using multivariable analysis, and overall survival (OS) was assessed with the inverse probability of treatment weighting propensity score.

**Results:**

A total of 3880 patients were examined in the National Cancer Database including 1829 women (median age, 56 [IQR, 49-63] years) with clear cell, 1156 women (median age, 53 [IQR, 42-64] years) with low-grade serous, and 895 women (median age, 57 [IQR, 48-66] years) with mucinous carcinomas. NACT use increased in patients with clear cell (from 10.2% to 16.2%, 58.8% relative increase; *P* < .001 for trend) or low-grade serous (from 7.7% to 14.2%, 84.4% relative increase; *P* = .007 for trend) carcinoma during the study period. This association remained consistent in multivariable analysis. NACT use also increased, but nonsignificantly, in mucinous carcinomas (from 8.6% to 13.9%, 61.6% relative increase; *P* = .07 for trend). Across the 3 histologic subtypes, older age and stage IV disease were independently associated with NACT use. In a propensity score–weighted model, the NACT and PDS groups had comparable OS for clear cell (4-year rates, 31.4% vs 37.7%; hazard ratio [HR], 1.12; 95% CI, 0.95-1.33) and mucinous (27.0% vs 26.7%; HR, 0.90; 95% CI, 0.68-1.19) carcinomas. For patients with low-grade serous carcinoma, NACT was associated with decreased OS compared with PDS (4-year rates, 56.4% vs 81.0%; HR, 2.12; 95% CI, 1.55-2.90). Increasing NACT use and histologic subtype–specific survival association were also found in the Surveillance, Epidemiology, and End Results Program cohort (n = 1447). A meta-analysis of 4 studies, including the current study, observed similar OS associations for clear cell (HR, 1.13; 95% CI, 0.96-1.34; 2 studies), mucinous (HR, 0.93; 95% CI, 0.71-1.21; 2 studies), and low-grade serous (HR, 2.11; 95% CI, 1.63-2.74; 3 studies) carcinomas.

**Conclusions and Relevance:**

Despite the lack of data on outcomes of NACT among patients with less common carcinomas, this study noted that NACT use for advanced disease has gradually increased in the US. Primary chemotherapy for advanced-stage, low-grade serous ovarian cancer may be associated with worse survival compared with PDS.

## Key Points

**Question:**

What are the use and outcomes of neoadjuvant chemotherapy (NACT) for treatment of less common epithelial ovarian cancer subtypes?

**Findings:**

In this cohort study of 3880 patients in the National Cancer Database with stage III to IV clear cell, low-grade serous, or mucinous ovarian cancers who received multimodal surgery and chemotherapy, NACT use increased in clear cell and low-grade serous carcinomas from 2006 to 2017. Patients with low-grade serous carcinoma who received NACT had lower overall survival rates than those receiving primary surgery, and the Surveillance, Epidemiology, and End Result Program cohort evaluation and meta-analysis supported the results.

**Meaning:**

The findings of this study suggest that there is a gradual increase in the number of patients receiving NACT for less common epithelial ovarian cancer in the US; NACT may be associated with poor survival outcomes in low-grade serous carcinomas.

## Teaser

This cohort study evaluates the use of neoadjuvant chemotherapy in women with less common epithelial ovarian carcinoma subtypes, including clear cell, mucinous, and low-grade serous cancers.

## Introduction

In 2023, ovarian cancer is expected to be the fifth most lethal female malignant neoplasm in the US.^1^ According to the American Cancer Society’s annual estimates, approximately 19 710 new diagnoses and 13 270 deaths due to ovarian cancer are predicted in 2023.^1^

Patients with ovarian cancer commonly present with advanced disease for which a multimodal strategy with cytoreductive surgery and systemic chemotherapy is the standard approach for primary treatment.^2,3^ The sequence of surgery and chemotherapy has evolved,^4,5^ and the paradigm has shifted toward neoadjuvant chemotherapy (NACT) for advanced ovarian cancer in parallel to the mounting evidence demonstrating survival outcomes comparable to those of primary debulking surgery (PDS).^6,7,8,9^

Ovarian cancer is heterogeneous, with distinct clinical and biological differences across its histologic subtypes.^10,11^ The most prevalent histologic subtype of epithelial ovarian cancer is high-grade serous carcinomas, and clear cell, low-grade serous, and mucinous carcinomas represent less common histologic subtypes in the US (2%-7%).^11^ Due to their rarity, the use and survival outcomes associated with NACT in these less common epithelial carcinomas have been relatively understudied.^6,8,12,13,14,15,16,17^ Even with the available data, limited sample size and lack of comparator groups make their findings difficult to interpret and adopt.

Phase 3 randomized clinical trials examining the effectiveness of NACT for advanced ovarian cancer have predominantly included patients with high-grade serous carcinomas.^6,7,8,9^ Clear cell (0%-8.2%), mucinous (0%-3.3%), and low-grade serous (0%-4.1%) carcinomas represented only small fractions in the 4 previous landmark studies (eTable 1 in Supplement 1).^6,7,8,9^ Thus, the survival effects of NACT in these less common histologic subtypes are not interpretable.

Moreover, current clinical practice guidelines do not specify the use of NACT for these less common histologic subtypes,^3,18^ and it is unknown whether the recent increase in NACT use for advanced ovarian cancer is also occurring in these less common carcinomas. The objective of the present study was to examine the uptake and survival outcomes of NACT for less common histologic subtypes of epithelial ovarian cancer.

## Methods

### Data Source

The current study used the National Cancer Database (NCDB) and the Surveillance, Epidemiology, and End Result (SEER) program. The NCDB is the comprehensive tumor registry including more than 1500 Commission on Cancer–accredited hospitals through a joint effort by the American College of Surgeons and the American Cancer Society.^19^ Approximately 70% of new invasive cancers are collected in the NCDB capturing schema. SEER, the National Cancer Institute tumor registry, has been in use for nearly 5 decades.^20^ SEER is a population-based database that captures nearly 97% of new incident cases within the registry area. The most recent version of the program covers about 35% of the US population. The University of Southern California Institutional Review Board waived the requirement for informed consent because the study included only publicly available, deidentified data. The Strengthening the Reporting of Observational Studies in Epidemiology (STROBE) reporting guideline and Preferred Reporting Items for Systematic Reviews and Meta-analyses (PRISMA) reporting guideline were consulted to outline the results of the observational cohort study and systematic review with meta-analysis study.

### Study Population

This retrospective cohort study with a systematic review and meta-analysis examined data from the NCDB from 2006-2017 and SEER from 2006-2019. The starting point of 2006 was chosen because this was when data became available regarding the sequence of cancer-directed surgery and systemic chemotherapy in both databases. The study population included patients with stage III to IV ovarian cancer with clear cell, mucinous, and low-grade serous histologic subtypes who received multimodal treatment with cancer-directed surgery and systemic chemotherapy. All cases had pathologic confirmation of the histologic diagnosis.

Histologic subtypes were based on the World Health Organization’s *International Classification of Diseases for Oncology, Third Edition*^21^: clear cell (codes 8005/3, 8310/3, 8313/3, 8443/3, and 8444/3), mucinous (codes 8470/3, 8471/3, 8472/3, 8480/3, 8481/3, 8482/3, and 9015/3), and low-grade serous (codes 8441/3, 8442/3, 8460/3, 8461/3, 8462/3, and 9014/3). This classification system defines low-grade serous carcinoma as serous histologic subtype that is well differentiated per previous studies.^11,22,23^ Exclusion criteria included nonmalignant cases, histologic type other than those listed above, no diagnostic confirmation, second primary cancer, stage I to II or unknown stage, no or unknown cancer-directed surgery, no or unknown chemotherapy, chemotherapy use during surgery, and unknown sequence for surgery and chemotherapy.

### Exposure

The exposure assignment was the sequence of treatment. Patients who underwent PDS followed by postoperative chemotherapy were assigned to the PDS group. Patients who received primary chemotherapy followed by interval surgery were assigned to the NACT group. The sequence information was available as a distinct variable (RX_SUMM_SYSTEMIC_SUR_SEQ) in both data sets.

### Outcome Measures

The co-primary outcomes were temporal trends and characteristics of NACT use and overall survival (OS) associated with NACT. Overall survival was defined as the time interval between the initial ovarian cancer diagnosis and death for any reason (all-cause). Patients alive at the last follow-up were censored.

### Study Covariates

Preselected study covariates included patient age (continuous), study period (quarterized as 3-year increments), race and ethnicity (Asian and Pacific Islander, Hispanic, Native American, non-Hispanic Black, and non-Hispanic White), insurance type (private, Medicaid, Medicare, and other), zip code–level median household income (every quartile per the 2000 Census), residence-level educational attainment for non–high school degrees (every quartile per the 2000 Census), and Charlson-Deyo Comorbidity Index score (0, 1, and ≥2).^24^ Race and ethnicity were included as this information is important for ovarian cancer characteristics.

Hospital-level information included facility type (community cancer program, comprehensive community cancer program, academic/research program, and integrated network cancer program) and facility location (East, Central North, Central South, and West). Tumor characteristics included cancer stage (IIIA, IIIB, IIIC, and IV) and CA125 status (negative/normal or positive/elevated) determined per the Collaborative Stage Site-Specific Factor 1.^25^ Surgical data included cytoreduction status (optimal debulking including ≤1-cm residual tumor nodule or suboptimal debulking including >1-cm residual tumor nodule) per Collaborative Stage Site-Specific Factor 3.^25^

### Statistical Analysis

Statistical analysis was conducted separately within the parallel main cohorts (NCDB and SEER). The first step of analysis was to evaluate temporal trends of the use of NACT during the study period. This step of analysis was assessed with the Cochran-Armitage test. Data analysis was performed from July 2022 to April 2023.

The second step of analysis was to examine the independent characteristics associated with NACT use. A binary logistic regression model with conditional backward method was fitted for multivariable analysis.^26^ Baseline preoperative study covariates with *P* < .05 in the univariable analysis were considered in the initial selection, and the least significant covariate was removed from the model until all covariates retained *P* < .05 in the final model. To avoid overfitting, this parsimonious modeling was predetermined under the assumption that the use of NACT is likely low to modest. Multicollinearity among the covariates was evaluated. Effect sizes for NACT use compared with PDS were calculated as adjusted odds ratios with corresponding 95% CIs.

The third step of analysis was to examine the association between primary treatment and OS, which was compared between the NACT and PDS groups from 2010 to 2016. The starting period was chosen due to the availability of intraoperative information for cytoreduction status, which is a key prognostic factor for ovarian cancer. The end period was chosen due to the availability of survival data in the NCDB.

Inverse probability of treatment weighting (IPTW) propensity score was used to mitigate the background difference between the 2 exposure groups.^27^ Stringent study covariates were preselected per historical view for the IPTW modeling due to the assumption that sample size is likely limited. Age, year, comorbidity score, CA125 status, cancer stage, and residual disease at surgery were considered in the IPTW modeling as these covariates were prognostic in ovarian cancer and also can be treatment decision factors for NACT use.^2,5^

The IPTW propensity score method assigned patients in the NACT group a weight of 1 / (propensity score) and those in the PDS group a weight of 1 / (1 − propensity score). Stabilized weights and threshold technique at 10 were used. Next, the IPTW cohort was created, and clinical size imbalance was calculated with standardized difference between the exposure groups. A value greater than 0.20 was interpreted as clinical imbalance between the exposure groups and informed for model adjusting.^28^ The 4-year OS rates were calculated with the Kaplan-Meier method for survival point estimates that were chosen based on the median follow-up of censored cases in the study cohort, and the effect size for NACT vs PDS was expressed with hazard ratios (HRs) and corresponding 95% CIs.

Various sensitivity analyses were performed to assess the robustness of the study findings. First, trends and survival outcomes were assessed based on the clinicopathologic relevance for exposure, including evaluation of age, tumor extent, and comorbidity. In addition, patients with unknown information were excluded from analysis.

The last step of the analysis was to perform a systematic literature review and meta-analysis to examine the survival associated with NACT for oncologic outcome in less common epithelial ovarian cancer. The details of inclusion and exclusion criteria and analytic approach are described in eMethods 1 and eMethods 2 in Supplement 1. Briefly, 3 public search engines (PubMed, Cochrane Database of Systematic Reviews, and Scopus) were used with the keywords *neoadjuvant therapy* [Medical Subject Heading] (except for Scopus) or *neoadjuvant* or *followed by interval debulking* or followed by cytoreduc* or *primary chemotherapy* executed in each histologic subtype (performed by S.M. and M.M.). The primary outcome measure was OS.

Statistical analyses were based on 2-tailed hypotheses, and *P* < .05 was considered statistically significant. Patients with missing information were grouped as one in each covariate. Analysis was conducted using SPSS Statistics, version 28.0 (IBM Corp) and R, version 3.5.3 (R Foundation for Statistical Computing). Pooled HRs were calculated using RevMan, version 5.4.1 (Cochrane Collaboration).

## Results

### NCDB Cohort

A total of 3880 patients met the eligibility criteria in the NCDB cohort (eFigure 1 in Supplement 1). The most frequent histologic subtype was clear cell (n = 1829), followed by low-grade serous (n = 1156) and mucinous (n = 895) carcinomas. The median age of the patients was 56 (IQR, 49-63) years for clear cell, 53 (IQR, 42-64) years for low-grade serous, and 57 (IQR, 48-66) years for mucinous carcinomas. Treatment with NACT was used in 253 patients (13.8%) with clear cell, 120 patients (10.4%) with low-grade serous, and 95 patients (10.6%) with mucinous carcinomas (eTable 2 in Supplement 1).

**Figure 1.**
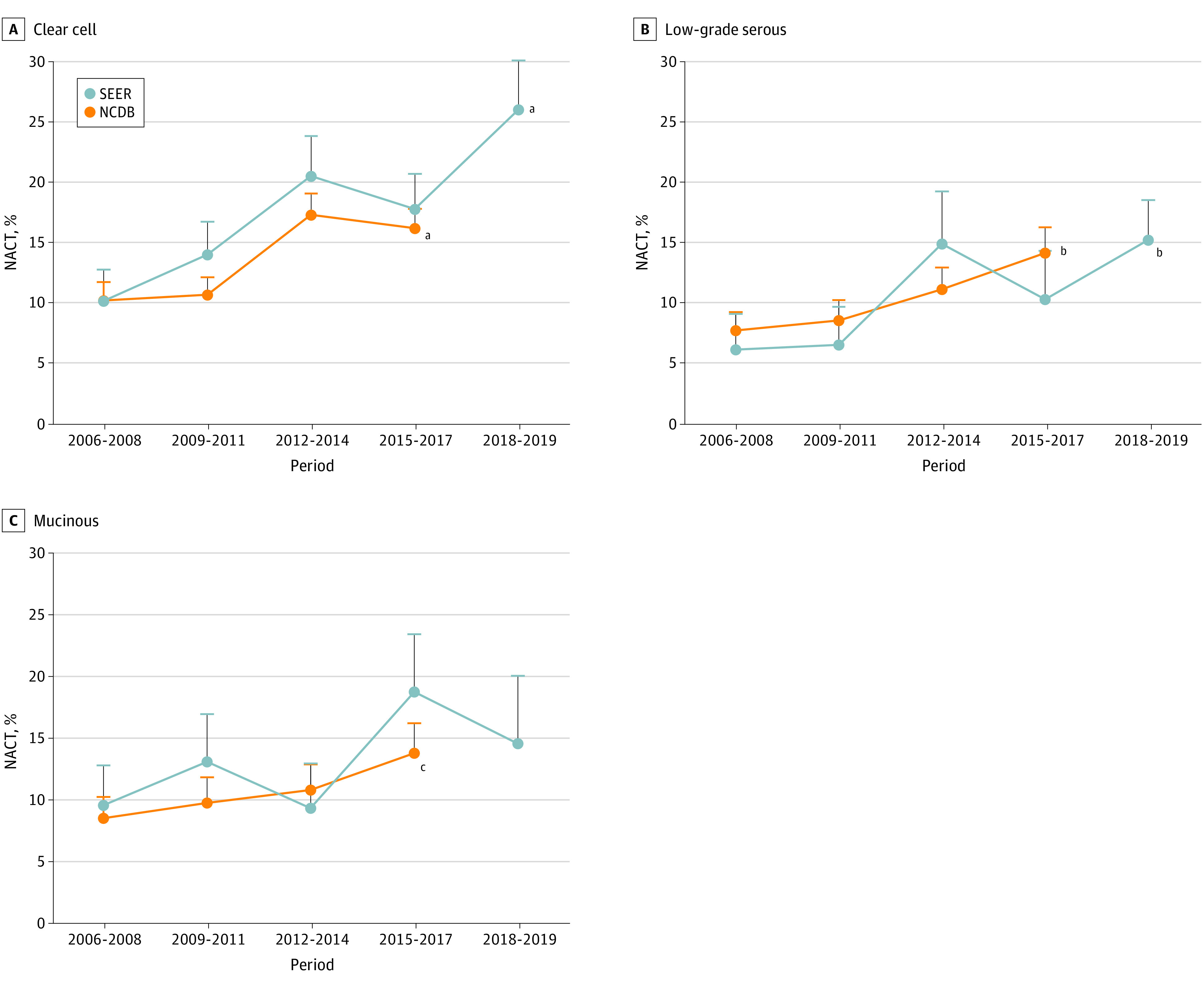
Temporal Trends Use of neoadjuvant chemotherapy (NACT) is shown in 3-year increments during the study period for clear cell (A), low-grade serous (B), and mucinous (C) carcinomas in the main study cohort (National Cancer Database [NCDB] 2006-2017) and the second study cohort (Surveillance, Epidemiology, and End Results [SEER] 2006-2019). Error bars represent SEs. The Cochran-Armitage trend test was used to determine *P* for trend values. ^a^*P* < .001 for trend. ^b^*P* < .05 for trend. ^c^*P* = .07 for trend.

From 2006 to 2017, NACT use increased in clear cell carcinomas from 10.2% to 16.2% (58.8% relative increase; *P* < .001 for trend) (Figure 1A), and in low-grade serous carcinomas from 7.7% to 14.2% (84.4% relative increase; *P* = .007 for trend) (Figure 1B). Albeit statistically nonsignificant, NACT use also increased in mucinous carcinomas from 8.6% to 13.9% (61.6% relative increase; *P* = .07) (Figure 1C). In analyses stratified by patient and tumor characteristics (eTable 3 in Supplement 1), the interval increase in NACT use was particularly high in young patients with clear cell carcinomas, older patients with low-grade serous or mucinous carcinomas, patients with minimal comorbidity and clear cell and low-grade serous carcinomas, patients with stage III disease and clear cell carcinomas, and patients with stage IV disease and low-grade serous or mucinous carcinomas.

In multivariable analysis (eTable 4 in Supplement 1), the increasing use of NACT during the study period remained independent for clear cell and low-grade serous carcinomas. In addition, across the 3 histologic subtypes, older age and stage IV disease were independently associated with NACT use.

Intraoperative performance of cytoreduction was examined (eTable 5 in Supplement 1). Across the 3 histologic subtypes, the rate of optimal cytoreduction was lower in the NACT group than in the PDS group (66.7% vs 75.3% for clear cell, 56.7% vs 75.5% for low-grade serous, and 52.3% vs 67.3% for mucinous carcinomas; all *P* < .001).

In a propensity score–based IPTW model (eFigure 2 in Supplement 1), all covariates were overall more balanced compared with the pre-IPTW model (standardized difference ≤0.186 for clear cell, ≤0.257 for low-grade serous, and ≤0.196 for mucinous carcinomas). The median follow-up of censored patients was 44.7 (IQR, 28.5-65.9) months for clear cell, 52.5 (IQR, 34.7-70.5) months for low-grade serous, and 43.1 (IQR, 27.8-61.4) months for mucinous carcinomas.

**Figure 2.**
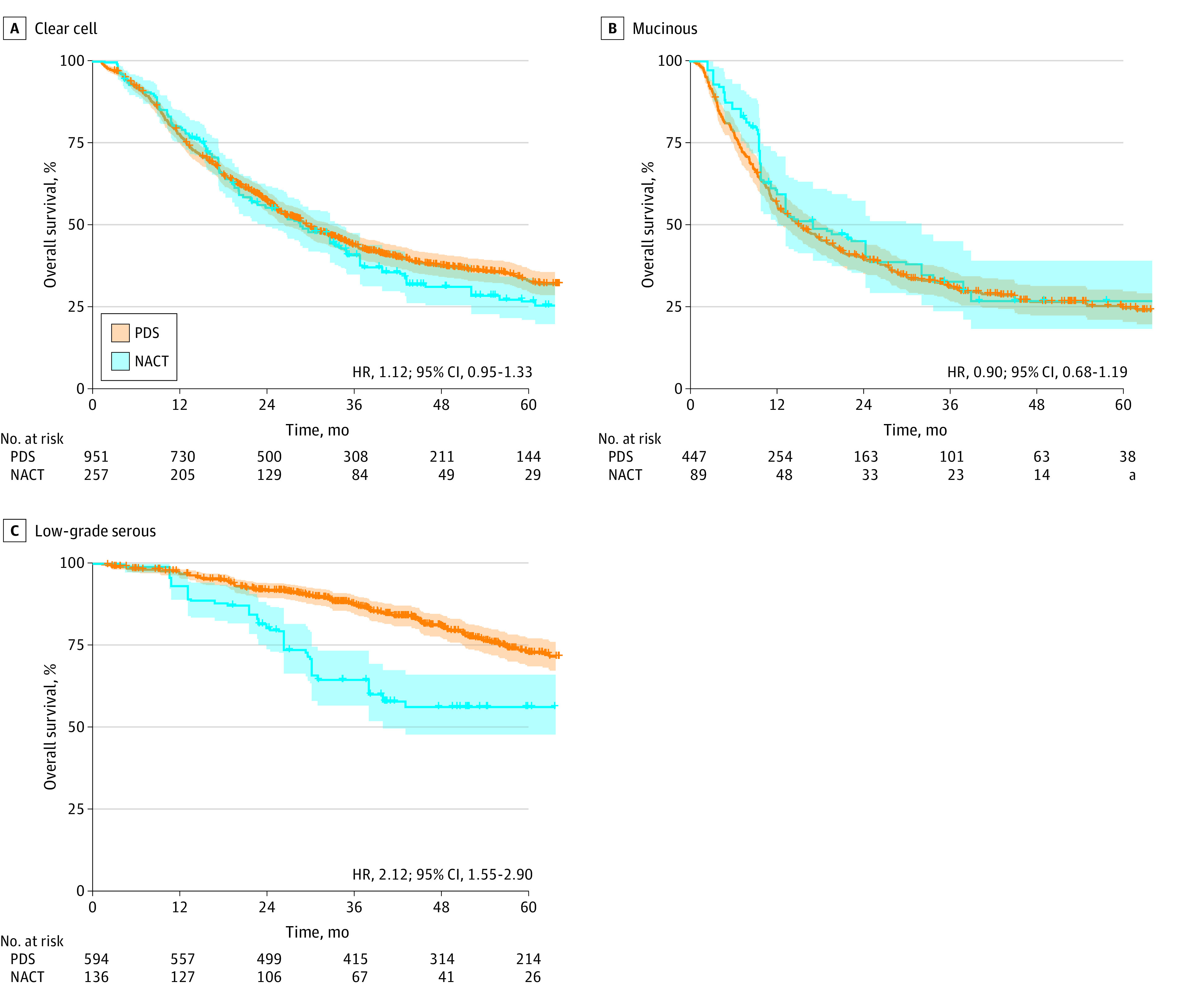
Overall Survival in the National Cancer Database Cohort Overall survival based on the exposure assignment (neoadjuvant chemotherapy [NACT] vs primary debulking surgery [PDS]) is shown for clear cell (A), mucinous (B), and low-grade serous (C) carcinomas. The survival curves were constructed in the inverse probability of treatment weighting cohorts in each histologic subtype. The exposure-outcome association was also found when unknown cases were excluded in the analysis. Color band widths indicate 95% CIs. Number at risk is per a propensity score–weighted model. HR indicates hazard ratio. ^a^Small number suppressed.

**Figure 3.**
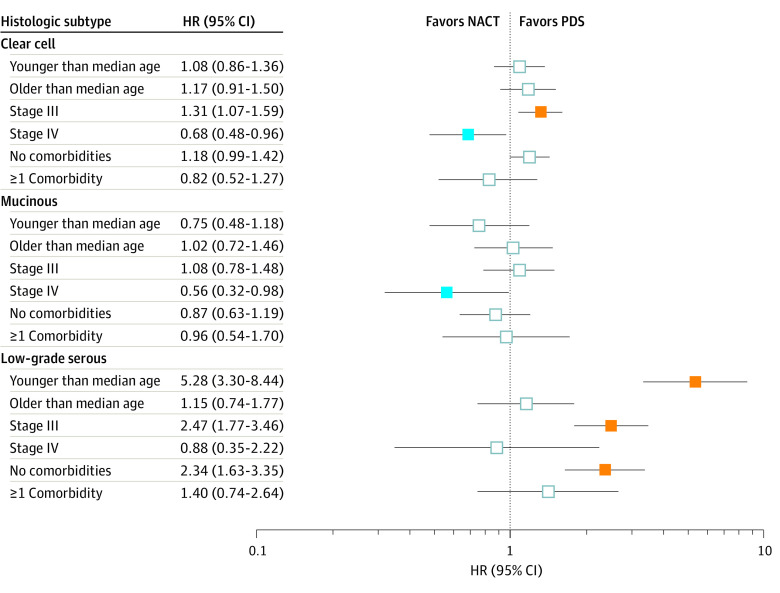
Subgroup Analysis of the National Cancer Database Cohort Overall survival associated with the exposure (neoadjuvant chemotherapy [NACT] compared with primary debulking surgery [PDS]) in each histologic subtype and the hazard ratio (HR) and the corresponding 95% CIs are displayed (orange boxes indicate HR>1 with *P* < .05; blue boxes indicate HR<1 with *P* < .05). Younger and older age groups were based on the median age of patients within each histologic subtype. Comorbidity was dichotomized by Charlson-Deyo score (0 vs ≥1). Analysis was based on the inverse probability of treatment weighting model.

The NACT and PDS groups had comparable OS for patients with clear cell (4-year rates, 31.4% vs 37.7%; HR, 1.12; 95% CI, 0.95-1.33) (Figure 2A) and mucinous (27.0% vs 26.7%; HR, 0.90; 95% CI, 0.68-1.19) (Figure 2B) carcinomas. For patients with low-grade serous carcinomas, the NACT group had a lower 4-year OS rate than the PDS group (56.4% vs 81.0%; HR, 2.12; 95% CI, 1.55-2.90) (Figure 2C). In the subcohort analyses (Figure 3), a marked effect size was observed in low-grade serous carcinomas, and patients in the NACT group who were younger, had lower comorbidity scores, and had a lesser extent of disease were more than twice as likely to die than their counterparts in the PDS group.

### SEER Cohort

In the evaluation using data from the SEER program (eFigure 3 in Supplement 1), a total of 1447 patients met the study eligible criteria. The most frequent tumor type was clear cell (n = 745), followed by low-grade serous (n = 369) and mucinous (n = 333).

Increasing NACT use was observed in clear cell (10.2% to 26.1%; 159% relative increase; *P* < .001 for trend) (Figure 1A) and low-grade serous (6.2% to 15.3%; 147% relative increase; *P* = .04 for trend) (Figure 1B) carcinomas from 2006 to 2019. The relative increase in the mucinous group was 52.1% (*P* = .20 for trend) (Figure 1C).

The NACT and PDS groups had comparable OS for patients with clear cell (HR, 0.93; 95% CI, 0.74-1.16) and mucinous (HR, 1.13; 95% CI, 0.72-1.78) carcinomas (eFigure 4A and B in Supplement 1). The NACT group had worse OS outcomes compared with the PDS group among patients with low-grade serous carcinomas (HR, 3.17; 95% CI, 1.57-6.40) (eFigure 4C in Supplement 1).

### Systematic Review and Meta-analysis

A systematic review of the literature identified 8 studies (eFigure 5 and eTable 6 in Supplement 1).^6,8,12,13,14,15,16,17^ Of those, 3 studies provided HRs for OS. Together with the NCDB cohort in the current study, a total of 4 studies were evaluated in our meta-analysis (eFigure 4 in Supplement 1).^6,15,17^

**Figure 4.**
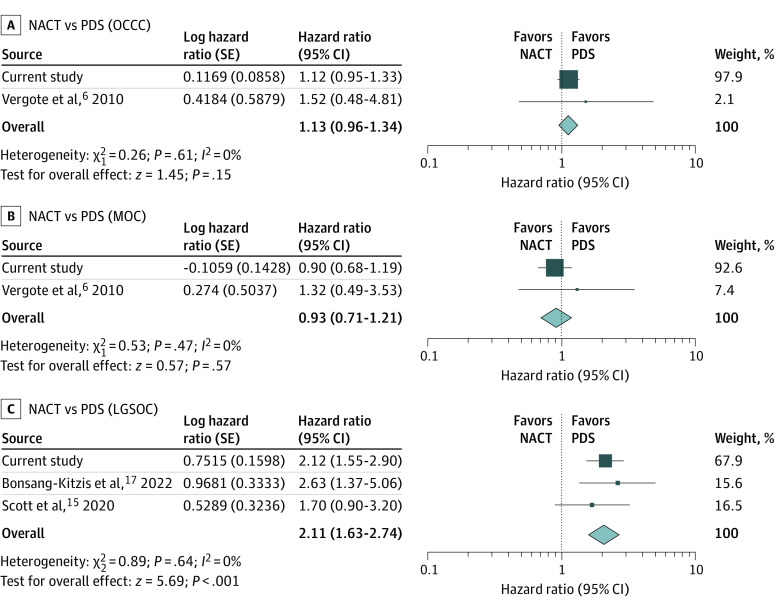
Meta-analysis of Neoadjuvant Chemotherapy (NACT) and Overall Survival Results of a meta-analysis for the association of neoadjuvant chemotherapy (NACT) with overall survival in patients with clear cell (A), mucinous (B), and low-grade serous (C) carcinomas are shown. Results of a fixed-effects meta-analysis of studies for overall survival are ordered within stratum by year of publication and relative weight (%) of studies. No heterogeneity was noted among the studies (*I^2^* = 0%). LGSOC indicates low-grade serous ovarian carcinoma; MOC, mucinous ovarian carcinoma; OCCC, ovarian clear cell carcinoma; and PDS, primary debulking surgery.

NACT was not associated with OS in patients with clear cell (HR, 1.13; 95% CI, 0.96-1.34; 2 studies) and mucinous (HR, 0.93; 95% CI, 0.71-1.21; 2 studies) carcinomas, but NACT was associated with worse OS outcomes in patients with low-grade serous carcinomas (HR, 2.11; 95% CI, 1.63-2.74; 3 studies) (Figure 4). This association remained similar when the SEER cohort of the current study was included (eFigure 6 in Supplement 1). In an analysis stratified according to cancer stage, NACT was associated with worse OS outcomes in patients with stage III clear cell and low-grade serous carcinomas (eFigure 7 in Supplement 1) and better OS outcomes in patients with stage IV clear cell carcinomas (eFigure 8 in Supplement 1).

## Discussion

Our key findings are, first, that NACT use has increased in advanced clear cell and low-grade serous ovarian cancers. Second, the survival outcome of NACT for patients with advanced disease varied based on histologic subtype and cancer stage. While some of the results in this study may be known, the importance in certain areas merits further discussion.

The increased trend of NACT use for patients with less common ovarian cancer and advanced disease was observed in both Commission on Cancer–affiliated hospital data and the National Cancer Institute’s population-based data, supporting the validity of our findings. Therefore, these data suggest that use of NACT for advanced, less common ovarian cancer has gradually increased in the US.

As noted in eTable 1 in Supplement 1, less common histologic subtypes composed only a small portion of earlier randomized clinical trials.^6,7,8,9^ Given that, to our knowledge, there is no level I evidence (prospective randomized clinical trials) confirming the effectiveness of NACT specific to these less common ovarian cancer types, the increasing use of NACT was somewhat unexpected.

The reason for the increase in NACT use in less common ovarian cancer subtypes was not assessed in this study, but it is likely multifactorial. The most compelling reason may be the influence of previous phase 3 randomized clinical trials.^6,7,8,9^ The comparable survival outcomes of those studies between NACT and PDS approaches may cause NACT to be favored by surgeons and patients who are not medically optimized for major abdominopelvic surgery. Whether the health care professional and patient knew that less common histologic subtypes represented a small portion of patients included in the earlier trials was not known in this study, warranting further investigation.^6,7,8,9^

Consistent with earlier studies with predominantly high-grade serous carcinomas, older age and stage IV disease were the most common patient and tumor factors for NACT use in this study of less common histologic subtypes.^4^ Another important finding was residual disease at cytoreduction. Overall, the NACT group had higher rates of large residual disease at the time of the operation compared with the PDS group in the NCDB cohort (eTable 5 in Supplement 1). This result differs from earlier studies that predominantly examined high-grade serous carcinomas.^6,7,8,9,29,30^

Several hypotheses might explain these observed findings. The first possibility is that patients in the NACT group may have had a higher disease burden and tumors in unresectable locations. The initial evaluation with systemic imaging and/or diagnostic laparoscopy may have triaged patients with these factors to initiate NACT. A 2018 prospective study reported that patients who received NACT due to unfavorable tumor resectability as assessed by diagnostic laparoscopy had higher rates of suboptimal cytoreduction, resulting in shorter survival, compared with those who proceeded to PDS with a favorable chance of resectability.^31^

The second possibility is chemoresistance in clear cell, low-grade serous, and mucinous carcinomas. The combination regimen of carboplatin and paclitaxel was used in previous clinical trials in which most tumors had serous histologic characteristics.^6,7,8,9^ However, some investigators have suggested that these less common ovarian cancers may have lower rates of chemotherapy response.^12,32,33^ As this study did not have information regarding chemotherapy type, additional study is warranted to assess this hypothesis. Likewise, generalizability of the NCDB cohort needs to be examined as the findings are slightly different from the SEER cohort.

As the survival outcome of NACT was previously not well examined in less common ovarian cancers based on the results of the systematic literature review in this study, particularly in clear cell and mucinous carcinomas, some of the current study’s results add new information to the literature. Two notable associations included possible adverse effects of NACT in low-grade serous carcinomas and stage-specific effects of NACT on survival.

Across the 2 cohorts in this study and 2 previous studies, NACT use for low-grade serous ovarian cancer was associated with worse OS outcomes compared with PDS.^15,17^ This survival association was particularly prominent in prognostically more favorable subgroups in general (younger, healthier patients and earlier disease). This age interaction may support a study reporting shorter survival in younger patients with low-grade serous carcinomas.^34^ That study team speculated the possible effects of estrogen/progesterone receptor activity in younger patients and hypothesized that younger patients undergoing NACT for 3 to 4 months have their ovaries in situ, and hormone receptor–expressing tumors may continue to grow until oophorectomy at interval cytoreduction.^34^

Another key finding was the survival outcome of NACT per cancer stage. Among clear cell carcinomas, NACT was associated with worse OS in stage III disease and better OS in stage IV disease (eFigures 6 and 7 in Supplement 1). As previous studies did not provide stage-specific outcomes, the results of the current study provide clinically meaningful insights. Our results in stage III disease may support the aforementioned speculations about tumor burden/resectability and chemoresistance. Conversely, our results in stage IV disease suggest chemosensitive tumors in this group. Thus, cautious interpretation of this finding is appropriate.

### Limitations

Key limitations in this study included unmeasured confounders including tumor burden and location at the initial diagnosis, patient performance status, and details of comorbidity, such as thromboembolic event, assessment of resectability using systemic imaging and diagnostic laparoscopy, chemotherapy detail (type, cycle, and toxic effects), genetic and molecular characteristics, and the shared decision-making process. All of these factors could affect the exposure-outcome association.

Selection bias is another limitation. For instance, patients who received NACT were older and had higher stage disease. While these factors were balanced in the IPTW modeling and further examined in subcohort analyses, this bias may not be eliminated in the analysis and needs to be recognized as a major drawback with cautious interpretation. The number of patients excluded due to unknown treatment sequence for surgery and chemotherapy was another selection bias in this study. Immortal time bias needs to be recognized due to lack of information on treatment initiation data.

Progression-free survival, treatment response, surgical complications, and quality of life are important outcome measures for this type of study, but data were not available. Also, the accuracy of the data was not assessable as central pathologic findings review was not performed. This is important in the mucinous group, in which some tumors may be of gastrointestinal origin.^35^ Low-grade serous carcinoma is another tumor in which the lack of central pathologic review may affect the accuracy of histologic findings. The value of the meta-analysis may be limited due to the small number of previous studies. In addition, this study only examined the US population, and generalizability to other regions was not evaluated. Similarly, some cases may be overlapped between the 2 databases used in this study, and validity of the second cohort is limited.

## Conclusions

A one-size-fits-all concept may not apply in ovarian cancer treatment due to the heterogeneity of the tumors in clinical and biological characteristics^10^; thus, thoughtful interpretation is warranted when extrapolating data derived from common subtypes to less common histologic subtypes. Given a growing enthusiasm for NACT use in less common ovarian carcinomas (Figure 1), developing clinical practice guidelines and consensus to address the role of NACT in less common histologic subtypes would be useful.

Evolving novel therapeutic approaches may also be necessary given treatment that is resistant to conventional chemotherapy in these less common histologic subtypes. One such example may target therapy focused on tumor biologic factors for low-grade serous carcinomas. In a recent pilot study, NACT with antiestrogenic therapy and a CDK4/6 inhibitor for unresectable, untreated stage III to IV low-grade serous carcinomas demonstrated an unprecedented response rate (clinical benefit rate, 80%).^36^ Further development in a larger study population is warranted.

While necessary to address the unmet needs of evidence, prospective trials to evaluate the effectiveness of NACT in less common ovarian cancer subtypes would be likely challenging to conduct due to their rare incidence. National and international collaborations could facilitate the development of such trials. Barring more data, careful patient selection and shared decision-making are recommended when NACT is considered in these less common carcinomas.

## Notes

Supplement 1. **eMethods 1.** Systematic Literature Review and Meta-analysis
eReferences 1
**eMethods 2.** Search Key Words **eTable 1.** Proportion of Rare Epithelial Carcinomas in Prior Randomized Trials
eReferences 2
**eTable 2.** Baseline Demographics per Histology Types (NCDB Cohort) **eTable 3.** Temporal Trends of NACT per Age and Stage (NCDB Cohort) **eTable 4.** Multivariable Analysis for NACT Utilization (NCDB Cohort) **eTable 5.** Residual Disease at Surgery **eTable 6.** Meta-data of Eligible Studies
eReferences 3
**eFigure 1.** Study Selection Schema (NCDB Cohort) **eFigure 2.** Balance Statistics for IPTW (NCDB Cohort) **eFigure 3.** Study Selection Schema (SEER Cohort) **eFigure 4.** Overall Survival (SEER Cohort) **eFigure 5.** Study Selection Scheme of the Systematic Literature Search **eFigure 6.** Meta-analysis (Exploratory) **eFigure 7.** Meta-analysis for Stage III Disease (Exploratory) **eFigure 8.** Meta-analysis for Stage IV Disease (Exploratory)

Supplement 2.
Data Sharing Statement
