# Supplementary material for: Uptake and Outcomes of Neoadjuvant Chemotherapy Among US Patients With Less Common Epithelial Ovarian Carcinomas
Source: JAMA Netw Open. 2023 Jun 16;6(6):e2318602. doi: 10.1001/jamanetworkopen.2023.18602 (PMC10276312; doi:10.1001/jamanetworkopen.2023.18602)
Supplement: Supplement 1. — eMethods 1. Systematic Literature Review and Meta-analysis eReferences 1 eMethods 2. Search Key Words eTable 1. Proportion of Rare Epithelial Carcinomas in Prior Randomized Trials eReferences 2 eTable 2. Baseline Demographics per Histology Types (NCDB Cohort) eTable 3. Temporal Trends of NACT per Age and Stage (NCDB Cohort) eTable 4. Multivariable Analysis for NACT Utilization (NCDB Cohort) eTable 5. Residual Disease at Surgery eTable 6. Meta-data of Eligible Studies eReferences 3 eFigure 1. Study Selection Schema (NCDB Cohort) eFigure 2. Balance Statistics for IPTW (NCDB Cohort) eFigure 3. Study Selection Schema (SEER Cohort) eFigure 4. Overall Survival (SEER Cohort) eFigure 5. Study Selection Scheme of the Systematic Literature Search eFigure 6. Meta-analysis (Exploratory) eFigure 7. Meta-analysis for Stage III Disease (Exploratory) eFigure 8. Meta-analysis for Stage IV Disease (Exploratory) [file jamanetwopen-e2318602-s001.pdf]

Supplementary Online Content

Matsuo K, Matsuzaki S, Maeda M, et al. Uptake and outcomes of neoadjuvant chemotherapy among US patients with less common epithelial ovarian carcinomas. *JAMA Netw Open*. 2023;6(6):e2318602. doi:10.1001/jamanetworkopen.2023.18602

eMethods 1. Systematic Literature Review and Meta-analysis

eReferences 1

eMethods 2. Search Key Words

eTable 1. Proportion of Rare Epithelial Carcinomas in Prior Randomized Trials

eReferences 2

eTable 2. Baseline Demographics per Histology Types (NCDB Cohort)

eTable 3. Temporal Trends of NACT per Age and Stage (NCDB Cohort)

eTable 4. Multivariable Analysis for NACT Utilization (NCDB Cohort)

eTable 5. Residual Disease at Surgery

eTable 6. Meta-data of Eligible Studies

eReferences 3

eFigure 1. Study Selection Schema (NCDB Cohort)

eFigure 2. Balance Statistics for IPTW (NCDB Cohort)

eFigure 3. Study Selection Schema (SEER Cohort)

eFigure 4. Overall Survival (SEER Cohort)

eFigure 5. Study Selection Scheme of the Systematic Literature Search

eFigure 6. Meta-analysis (Exploratory)

eFigure 7. Meta-analysis for Stage III Disease (Exploratory)

eFigure 8. Meta-analysis for Stage IV Disease (Exploratory)

This supplementary material has been provided by the authors to give readers additional information about their work.

© 2023 Matsuo K et al. *JAMA Network Open.*

eMethods 1. Systematic literature review and meta-analysis.

A systematic review (PROSPERO registration ID: CRD42022359329) and meta-analysis was conducted to determine the effect of neoadjuvant chemotherapy (NACT) on overall survival [OS] and disease-free survival [DFS] among patients with low-grade ovarian serous carcinoma (LGSOC), ovarian clear cell carcinoma (OCCC), and mucinous ovarian cancer (MOC). The meta-analysis investigated survival outcomes by comparing the patients treated with primary debulking surgery and NACT.

Article retrieval
We conducted a systematic search of articles published through July 31, 2022, using PubMed, Scopus, and Cochrane Library as performed in our previous study.[1-3] We reviewed articles according to the Preferred Reporting Items for Systematic Reviews and Meta-Analyses guidelines.[4] Studies were identified by screening the titles, abstracts, and full texts of relevant articles, as previously described.[1-3] All titles and abstracts were screened by Shinya Matsuzaki and Michihide Maeda.

Initially, various patterns of keywords listed in Supplementary Methods S2 were used to identify studies on ovarian cancer. Thereafter, the selected articles were screened to identify studies that examined the effect of NACT for patients with LGSOC, OCCC, and MOC, using the following keywords: Neoadjuvant therapy [MeSH] (except for Scopus) OR Neoadjuvant OR "followed by interval debulking" OR "followed by cytoreduc*" OR "primary chemotherapy"

Inclusion criteria
Studies were included if they met the following inclusion criteria: (*i*) patients with LGSOC, OCCC, and MOC (treated with NACT); (*ii*) sufficient information about NACT and relevant outcomes; (*iii*) specific relevant outcomes in patients with LGSOC, OCCC, and MOC were clarified; (*iv*) original articles involving studies, such as retrospective or prospective cohort studies, population-based studies, case-control studies, and randomized controlled trials.

Exclusion criteria
The studies with following criteria were excluded: (*i*) insufficient information regarding NACT; (*ii*) insufficient survival or recurrence information; (*iii*) not in the field of interest; (*iv*) articles involving case reports, case series, and systematic reviews; (*v*) conference abstracts; and (*vi*) articles not in English.

Data extraction
Data were extracted and the following variables were recorded by Shinya Matsuzaki and Michihide Maeda: first author's name, year of study, histology type, number of included cases, number of patients with primary debulking surgery, number of patients with NACT, and outcomes of interest (OS and DFS).

Meta-analysis plan
After the eligible studies were identified in the systematic literature review, the results of main study cohort (National Cancer Database) in the current study was added in the meta-analysis. Then, the results of second cohort (Surveillance, Epidemiology, and End Results Program) were further added as an exploratory fashion. This approach was undertaken because both databases were derived in the U.S. centers that certain cases may be possibly captured in the two mechanisms. Then, among the studies including the current two cohorts, stage-specific analysis (stage III and IV) was undertaken.

Survival outcome estimates for primary debulking surgery vs NACT were computed using the 95% confidence intervals of the reported values to estimate the hazard ratios for OS and DFS. Heterogeneity among the eligible studies was determined using I^2, which measures the percentage of total variation across studies. The meta-analysis and the production of all graphics were performed using RevMan 5.4.1 software (Cochrane Collaboration, Copenhagen, Denmark). For consistency, data from all outcomes (continuous and bivariate) were entered into RevMan 5.4.1 in such a way that negative effect sizes or relative risks <1 favored active intervention.

© 2023 Matsuo K et al. *JAMA Network Open.*

eReferences 1

1. Matsuzaki S, Matsuzaki S, Chang EJ, Yasukawa M, Roman LD, Matsuo K. Surgical and oncologic outcomes of hyperthermic intraperitoneal chemotherapy for uterine leiomyosarcoma: A systematic review of literature. Gynecologic oncology 2021;161:70-77.
2. Matsuzaki S, Klar M, Matsuzaki S, Roman LD, Sood AK, Matsuo K. Uterine carcinosarcoma: Contemporary clinical summary, molecular updates, and future research opportunity. Gynecologic oncology 2021;160:586-601.
3. Matsuo K, Novatt H, Matsuzaki S, et al. Wait-time for hysterectomy and survival of women with early-stage cervical cancer: A clinical implication during the coronavirus pandemic. Gynecologic oncology 2020;158:37-43.
4. Page MJ, McKenzie JE, Bossuyt PM, et al. Updating guidance for reporting systematic reviews: development of the PRISMA 2020 statement. Journal of clinical epidemiology 2021;134:103-12.

© 2023 Matsuo K et al. *JAMA Network Open.*

eMethods 2. Search keywords.

Three public searching engines used for analysis: PubMed, Scopus, and Cochrane.

1. PubMed
#1 Ovarian Neoplasms [MeSH]
#2 Pelvic Neoplasms [MeSH]
#3 Fallopian Tube Neoplasms [MeSH]
#4 Adnexal Diseases [MeSH]
#5 ovary [tiab] OR ovaries [tiab] OR ovarian [tiab] OR adnexa [tiab] OR fallopian [tiab] OR peritoneal [tiab]
#6 neoplasm [tiab] OR cancer [tiab] OR cancers [tiab] OR carcinoma [tiab] OR carcinomas [tiab] OR malignan* [tiab] OR tumor* [tiab] OR tumour* [tiab]
#7 #5 AND #6
#8 #1 OR #2 OR #3 OR #4 OR #7
#9 Neoadjuvant therapy [MeSH]
#10 Neoadjuvant [tiab] OR "followed by interval debulking" [tiab] OR "followed by cytoreduc*" [tiab] OR "primary chemotherapy" [tiab]
#11 #9 OR #10
#12 #8 AND #11
#13 "clear cell" [tiab] OR mucinous [tiab] OR low-grade [tiab] OR "rare type" [tiab] OR "chemo resistant" [tiab] OR "chemoresistance" [tiab]
#14 #12 AND #13

2. Scopus
#1 TITLE-ABS-KEY (ovarian OR ovary OR ovaries OR adnexa OR peritoneal OR fallopian) W/2 (neoplasm OR cancer OR malignan* OR tumor OR tumour OR carcinoma)
#2 TITLE-ABS-KEY (Neoadjuvant OR "followed by cytoreductive surgery" OR "followed by interval debulking" OR "primary chemotherapy" OR "before surgery")
#3 #1 AND #2
#4 TITLE-ABS-KEY (clear cell OR mucinous OR low-grade OR "rare type" OR "chemo resistant" OR "chemoresistance")
#5 #3 AND #4

3. Cochrane
#1 MeSH descriptor: [Ovarian Neoplasms]
#2 MeSH descriptor: [Adnexal Diseases]
#3 MeSH descriptor: [Fallopian Tube Neoplasms]
#4 MeSH descriptor: [Pelvic Neoplasms]
#5 (ovar* or adnexal or fallopian or peritoneal or pelvic) near/3 (cancer* or carcinoma* or malignan* or mass or masses or cyst or cysts or neoplasm* or tumor* or tumour*)
#6 (epithelial) near/5 (ovar*)
#7 #5 AND #6
#8 #1 OR #2 OR #3 OR #4 OR #7
#9 MeSH descriptor: [Neoadjuvant Therapy]
#10 #8 AND #9

© 2023 Matsuo K et al. *JAMA Network Open.*

eTable 1. Proportion of rare epithelial carcinomas in prior randomized trials.

Trial	EORTC-55971[1]		CHORUS[2]		SCORPION[3]		JCOG-0602[4]	
Year	2010		2015		2020		2020	
Exposure	PDS	NACT	PDS	NACT	PDS	NACT	PDS	NACT
No. patients (%)	*n*=336	*n*=334	*n*=255	*n*=219	*n*=84	*n*=87	*n*=147	*n*=130
Clear cell	6 (1.8)	4 (1.2)	4 (1.6)	13 (5.9)	1 (1.2)	0	12 (8.2)	4 (3.1)
Mucinous	8 (2.4)	11 (3.3)	2 (0.8)	4 (1.8)	0	0	2 (1.4)	2 (1.5)
Low-grade serous	n/a	n/a	10 (3.9)	9 (4.1)	1 (1.2)	0	n/a	n/a

Proportions of rare epithelial ovarian cancer (clear cell, mucinous, and low-grade serous) are shown per treatment type in each trial. *n/a*, not applicable; *PDS*, primary debulking surgery; and *NACT*, neoadjuvant chemotherapy.

© 2023 Matsuo K et al. *JAMA Network Open.*

eReferences 2

1. Vergote I, Tropé CG, Amant F, et al. Neoadjuvant chemotherapy or primary surgery in stage IIIC or IV ovarian cancer. The New England journal of medicine 2010;363:943-53.
2. Kehoe S, Hook J, Nankivell M, et al. Primary chemotherapy versus primary surgery for newly diagnosed advanced ovarian cancer (CHORUS): an open-label, randomised, controlled, non-inferiority trial. Lancet. 2015;386(9990):249-257.
3. Fagotti A, Ferrandina MG, Vizzielli G, et al. Randomized trial of primary debulking surgery versus neoadjuvant chemotherapy for advanced epithelial ovarian cancer (SCORPION-NCT01461850). Int J Gynecol Cancer. 2020;30(11):1657-1664.
4. Onda T, Satoh T, Ogawa G, et al. Comparison of survival between primary debulking surgery and neoadjuvant chemotherapy for stage III/IV ovarian, tubal and peritoneal cancers in phase III randomised trial. European journal of cancer (Oxford, England : 1990) 2020;130:114-25.

© 2023 Matsuo K et al. *JAMA Network Open.*

eTable 2. Baseline demographics per histology types (NCDB cohort).

	Clear cell			Low-grade serous			Mucinous		
Characteristic	PDS	NACT	*P*-value	PDS	NACT	*P*-value	PDS	NACT	*P*-value
No. patients	*n*=1,576	*n*=253		*n*=1,036	*n*=120		*n*=800	*n*=95	
Age	56 (49-63)	59 (52-67)	<.001	52 (41-63)	61 (50-71)	<.001	56 (47-65)	61 (53-72)	<.001
Year			<.001			.007			.07
1st quartile	22.3	15.8		26.4	19.2		30.6	24.2	
2nd quartile	26.0	19.4		24.6	20.0		23.0	21.1	
3rd quartile	23.9	31.2		26.2	28.3		24.6	25.3	
4th quartile	27.9	33.6		22.8	32.5		21.8	29.5	
Race/ethnicity			.59			.61			.61
Non-Hispanic White	79.9	77.1		84.0	86.7		74.5	77.9	
Other*	19.7	22.5		15.5	15.0		24.9	22.1	
Unknown	**	**		**	**		**	0	
Comorbidity index			.02			.06			.83
0	85.1	81.8		82.6	75.0		81.5	78.9	
1	12.8	13.0		14.7	19.2		13.8	15.8	
≥2	2.1	5.1		2.7	**		4.8	**	
Insurance			<.001			<.001			.07
Private	68.0	53.8		61.2	34.2		48.5	51.6	
Medicaid	6.3	11.1		9.7	14.2		13.3	**	
Medicare	18.5	29.6		20.8	40.0		27.9	35.8	
Other	5.8	4.3		6.2	**		9.0	**	
Unknown	1.4	**		2.0	**		1.4	0	
Median household income			.08			.03			.15
QT1 (lowest)	8.8	10.3		10.4	10.8		13.9	**	
QT2	12.2	18.2		14.2	21.7		17.4	22.1	
QT3	24.7	20.9		26.4	33.3		26.8	26.3	
QT4 (highest)	45.4	42.3		37.1	25.8		35.1	29.5	
Unknown	8.8	8.3		12.0	**		6.9	12.6	
No high school degree			.08			.03			.15
≥29%	8.8	10.3		10.4	10.8		13.9	**	
20.0-28.9%	12.2	18.2		14.2	21.7		17.4	22.1	
14.0-19.9%	24.7	20.9		26.4	33.3		26.8	26.3	
<14.0%	45.4	42.3		37.1	25.8		35.1	29.5	
Unknown	8.8	8.3		12.0	**		6.9	12.6	

© 2023 Matsuo K et al. *JAMA Network Open.*

Cancer stage			<.001			<.001			<.001
IIIA	9.9	7.5		9.9	**		9.6	0	
IIIB	8.3	5.1		10.4	**		10.5	**	
IIIC	60.2	39.9		63.2	51.4		51.1	37.9	
III NOS	2.9	**		3.6	**		3.6	**	
IV	18.7	45.5		12.8	36.7		25.1	49.5	
CA125			<.001			.07			.10
Negative/normal	7.6	**		8.6	**		9.1	**	
Positive/elevated	74.7	87.4		71.6	75.8		67.8	77.9	
Unknown	17.8	11.5		19.8	21.7		23.1	17.9	
Facility type			0.02			.10			.01
Community center	3.8	4.7		3.0	**		6.0	**	
Comprehensive community	34.6	26.9		26.0	30.0		36.5	44.2	
Academic/research	43.5	51.8		36.8	44.2		33.3	42.1	
Integrated network	12.3	13.8		12.4	11.7		11.4	**	
Unknown	5.8	**		21.9	13.3		12.9	**	
Facility location			0.78			.01			.21
East	41.4	41.1		42.0	32.7		39.5	31.5	
Central North	28.5	29.7		27.6	21.2		26.3	25.0	
Central South	11.9	9.8		17.4	28.8		18.4	19.6	
West	18.3	19.5		13.0	17.3		15.9	23.9	

Median (IQR) or percentage per group is shown. * Asian including Pacific Islanders, Hispanic, Native American, non-Hispanic Black, and other determined by the program. ** Small number suppressed (1-10). *PDS,* primary debulking; *NACT*, neoadjuvant chemotherapy.

© 2023 Matsuo K et al. *JAMA Network Open.*

eTable 3. Temporal trends of NACT per age and stage (NCDB cohort).

Characteristic	T1	T2	T3	T4	Δ (%)*	*P*-value
**Age stratification**						
Clear cell						
≤56 y	5.2	8.6	16.2	13.0	154.9	.001
>56 y	15.2	13.0	18.6	19.3	27.0	.10
Low-grade serous						
≤53 y	6.0	5.9	7.0	6.3	5.0	.80
>53 y	9.6	11.8	14.7	22.6	135.4	.002
Mucinous						
≤57 y	7.5	8.7	6.8	8.6	14.7	.91
>57 y	9.6	10.9	15.5	19.6	104.2	.02
**Comorbidity index**						
Clear cell						
0	9.4	10.2	16.9	16.0	70.2	<.001
≥1	14.8	13.5	20.0	17.4	17.6	.48
Low-grade serous						
0	6.5	6.3	11.9	13.2	103.1	.003
≥1	13.5	17.2	7.7	18.8	39.3	.82
Mucinous						
0	7.4	10.0	11.3	13.4	81.1	.05
≥1	13.5	8.8	9.0	15.8	17.0	.85
**Stage stratification**						
Clear cell						
Stage III	7.3	7.3	12.1	11.7	60.3	.01
Stage IV	22.7	22.5	37.6	28.8	26.9	.15
Low-grade serous						
Stage III	4.4	7.7	8.0	11.3	156.8	.008
Stage IV	25.0	15.2	30.2	26.4	5.6	.58
Mucinous						
Stage III	7.0	6.3	8.4	7.8	11.4	.63
Stage IV	12.0	17.7	18.5	32.7	172.5	.006

NACT utilization is shown in percentage per time period (3-year increments: 2006-2008 for T1, 2009-2011 for T2, 2012-2014 for T3, and 2015-2017 for T4). Age cutpoint was based on the median of each histology type. * Relative increase is the interval percentage change from T1 to T4. The Cochrane-Armitage trend test was used for *P*-values.

© 2023 Matsuo K et al. *JAMA Network Open.*

eTable 4. Multivariable analysis for NACT utilization (NCDB cohort).

	Clear cell		Low-grade serous		Mucinous	
Characteristic	aOR (95%CI)	*P*-value	aOR (95%CI)	*P*-value	aOR (95%CI)	*P*-value
Age	1.03 (1.02-1.04)	<.001	1.03 (1.02-1.05)	<.001	1.03 (1.01-1.05)	<.001
Year (quartile)	1.21 (1.06-1.37)	.003	1.26 (1.06-1.50)	.01		
Cancer stage						
III	1.00 (reference)		1.00 (reference)		1.00 (reference)	
IV	3.51 (2.64-4.66)	<.001	3.92 (2.57-5.99)	<.001	2.98 (1.92-4.61)	<.001
CA125		<.001*				
Negative/normal	0.15 (0.05-0.46)	.001				
Positive/elevated†	1.00 (reference)					
Unknown	0.56 (0.37-0.85)	.006				

A binary logistic regression model with conditional backward method (initial selection at *P*<.05 and stopping rule of *P*<.05) was fitted in each histology type, and only the covariates retained in the final model are displayed. *Overall *P*-value. †including borderline. *NACT*, neoadjuvant chemotherapy; *aOR*, adjusted odds ratio; *CI*, confidence interval.

© 2023 Matsuo K et al. *JAMA Network Open.*

eTable 5. Residual disease at surgery.

	Clear cell			Low-grade serous			Mucinous		
Outcome	PDS	NACT	*P*-value	PDS	NACT	*P*-value	PDS	NACT	*P*-value
NCDB cohort			<.001			<.001			<.001
Optimal	75.3	66.7		75.5	56.7		67.3	52.3	
Suboptimal	11.5	24.1		10.0	31.1		14.2	32.3	
Unknown	13.2	9.2		14.5	12.2		18.5	15.4	
SEER cohort			.04			.09			.82
Optimal	72.6	70.8		78.8	76.3		71.3	71.0	.
Suboptimal	10.8	18.9		8.6	18.4		15.9	19.4	
Unknown	16.6	10.4		12.7	5.3		12.8	9.7	

Percentage per exposure is shown. Examined the cases of 2010 or later due to the availability of information (Collaborative Stage Site-Specific Factor 3). Optimal included residual tumor nodule(s) ≤1cm, optimal debulking (size not given), and no gross residual disease. Suboptimal included residual tumor nodule(s) >1cm and macroscopic residual tumor (size not given). *PDS*, primary debulking surgery; *NACT*, neoadjuvant chemotherapy.

© 2023 Matsuo K et al. *JAMA Network Open.*

eTable 6. Meta-data of eligible studies.

Author	Year	Total	PDS	NACT	HGSOC	OCCC	MOC	LGSOC	Comparison	DFS (HR: 95%CI)	OS (HR: 95%CI)
Matsuo (NCDB)§	2023	1101	932	169	0	1101	0	0	NACT vs PDS (OCCC)	--	1.12 (0.95-1.33)
		497	442	55	0	0	497	0	NACT vs PDS (MOC)	--	0.90 (0.68-1.19)
		655	584	71	0	0	0	655	NACT vs PDS (LGSOC)	--	2.12 (1.55-2.90)
Matsuo (SEER)§	2023	558	452	106	0	558	0	0	NACT vs PDS (OCCC)	--	0.93 (0.74-1.16)
		226	195	31	0	0	226	0	NACT vs PDS (MOC)	--	1.13 (0.72-1.78)
		283	245	38	0	0	0	283	NACT vs PDS (LGSOC)	--	3.17 (1.57-6.40)
Bonsang[1]	2022	105	62	43	0	105	0	0	NACT vs PDS (LGSOC)	1.66 (1.03-2.69)	2.64 (1.37-5.06)
Cobb[2]	2020	72	0	72	36	36	0	0	HGSOC vs LGSOC (NACT)	#	††
Onda[3]	2020	20	14	6	--	--	16	4	NACT vs PDS (OCCC+MOC)	--	1.95 (0.72-5.34)
Scott[4]	2020	134	98	36	0	0	0	0	NACT vs PDS (LGSOC)	--	1.7 (0.9-3.2)
Chung[5]	2019	136	0	136	--	30	28	0	Others† vs OCCC (NACT)	2.10 (1.30-3.42)	2.62 (1.37-4.99)
		128	0	128	--	30	0	20	Others† vs MOC (NACT)	2.64 (1.54-4.50)	4.69 (2.48-8.85)
Kang[6]	2011	314	220	94	256	--	¶	¶	HGSOC vs OCCC+MOC (NACT)	1.08 (0.77-1.53)	--
Vergote[7]	2010	10	6	4	--	--	10	0	NACT vs PDS (OCCC)	--	1.52(0.48-4.81)‡
		19	8	11	--	--	0	19	NACT vs PDS (MOC)	--	1.32 (0.49-3.53)‡
Schmeler[8]	2008	25	0	25	0	25	0	0	LGSOC (no comparison)	*	**

Some values were inferred by the authors; thus, certain numbers in the table may be slightly different from the original values. §: present study; #: 16.4 months vs 18.5 months (*P*=0.69); ††: 47.4 months vs 48.2 months (*P*=0.85); ¶: the combined number of patients with OCCC and MOC was 58; *: 21.4 months; **: 56.1 months; †: LGSOC, endometroid carcinoma, undifferentiated and carcinosarcoma, etc. (*n*=106); ‡: estimated by the authors with reference to the image in Supplementary Figure S7. *NCDB*, National Cancer Database; *SEER*, Surveillance Epidemiology and End Results; *Total*, total number of eligible patients that met the relevant outcomes of interest; *PDS*, number of patients with primary debulking surgery; *NACT*, number of patients with neoadjuvant chemotherapy; *HGSOC*, high-grade serous ovarian carcinoma; *LGSOC*, low-grade ovarian serous carcinoma; *OCCC*, ovarian clear cell carcinoma; *MOC*, mucinous ovarian cancer; *DFS*, disease-free survival; *OS*, overall survival; *HR*, hazard ratio; *CI*, confidence interval; --, not applicable.

© 2023 Matsuo K et al. *JAMA Network Open.*

eReferences 3

1. Bonsang-Kitzis H, Panchbhaya N, Bats AS, et al. Surgical Implications of Advanced Low-Grade Serous Ovarian Cancer: Analysis of the Database of the Tumeurs Malignes Rares Gynécologiques Network. Cancers 2022;14.
2. Cobb LP, Sun CC, Iyer R, et al. The role of neoadjuvant chemotherapy in the management of low-grade serous carcinoma of the ovary and peritoneum: Further evidence of relative chemoresistance. Gynecologic oncology 2020;158:653-58.
3. Onda T, Satoh T, Ogawa G, et al. Comparison of survival between primary debulking surgery and neoadjuvant chemotherapy for stage III/IV ovarian, tubal and peritoneal cancers in phase III randomised trial. European journal of cancer (Oxford, England : 1990) 2020;130:114-25.
4. Scott SA, Llaurado Fernandez M, Kim H, et al. Low-grade serous carcinoma (LGSC): A Canadian multicenter review of practice patterns and patient outcomes. Gynecologic oncology 2020;157:36-45.
5. Chung YS, Park SY, Lee JY, et al. Outcomes of non-high grade serous carcinoma after neoadjuvant chemotherapy for advanced-stage ovarian cancer: a Korean gynecologic oncology group study (OV 1708). BMC cancer 2019;19:341.
6. Kang S, Kim TJ, Seo SS, Kim BG, Bae DS, Park SY. Interaction between preoperative CA-125 level and survival benefit of neoadjuvant chemotherapy in advanced epithelial ovarian cancer. Gynecologic oncology 2011;120:18-22.
7. Vergote I, Tropé CG, Amant F, et al. Neoadjuvant chemotherapy or primary surgery in stage IIIC or IV ovarian cancer. The New England journal of medicine 2010;363:943-53.
8. Schmeler KM, Sun CC, Bodurka DC, et al. Neoadjuvant chemotherapy for low-grade serous carcinoma of the ovary or peritoneum. Gynecologic oncology 2008;108:510-4.

© 2023 Matsuo K et al. *JAMA Network Open.*

eFigure 1. Study selection schema (NCDB cohort).

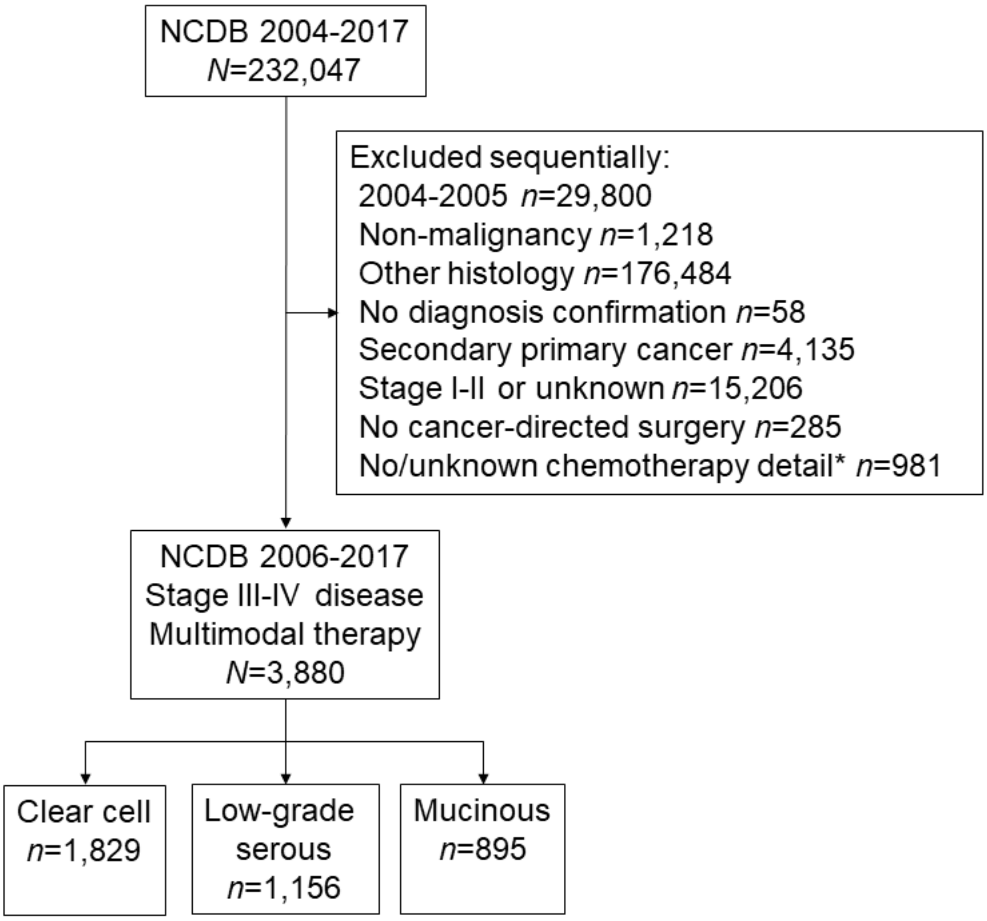

Multimodal therapy refers to cancer-directed surgery and systemic chemotherapy. * including unknown sequence of chemotherapy and cancer-directed surgery and intraperitoneal chemotherapy cases. *NCDB*, National Cancer Database.

© 2023 Matsuo K et al. *JAMA Network Open.*

eFigure 2. Balance statistics for IPTW (NCDB cohort).

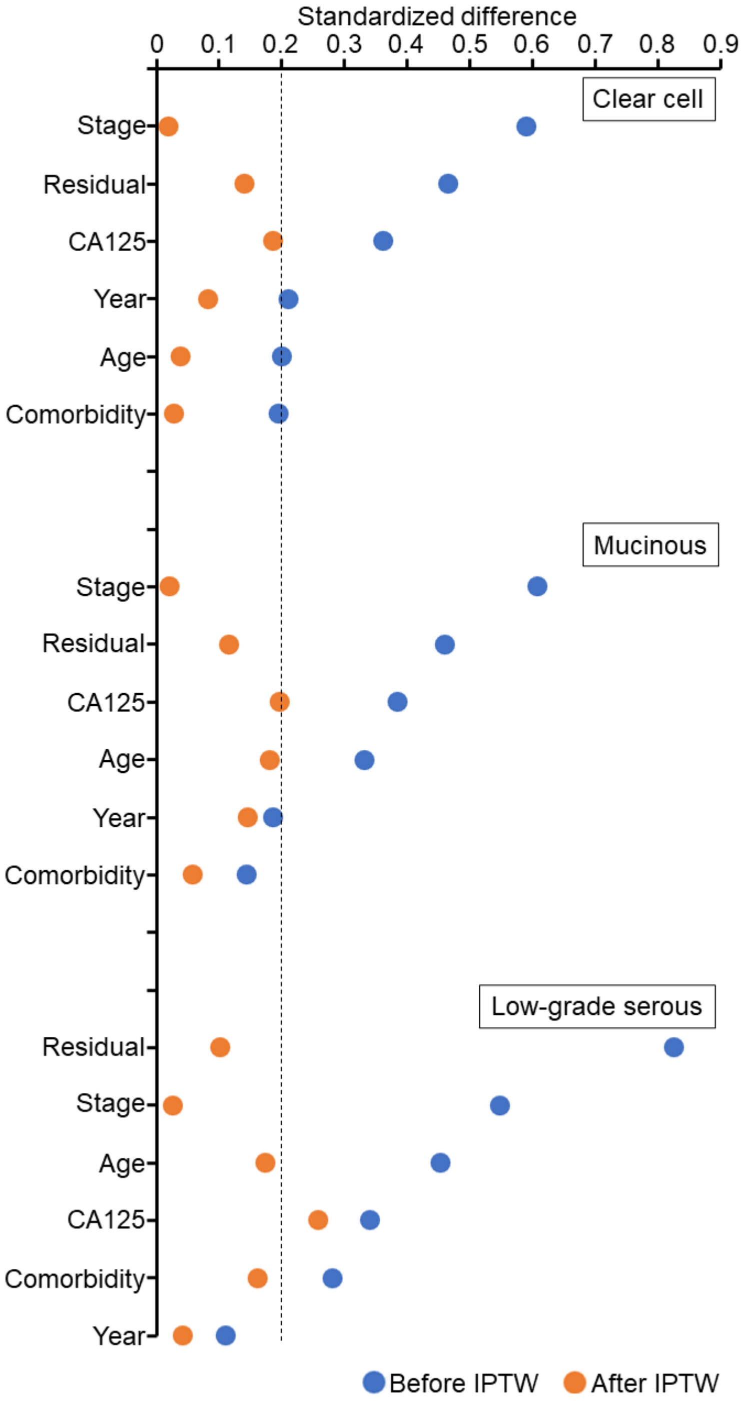

Vertical dashed line indicates the value of 0.20. Abbreviation: IPTW, inverse probability of treatment weighting.

© 2023 Matsuo K et al. *JAMA Network Open.*

eFigure 3. Study selection schema (SEER cohort).

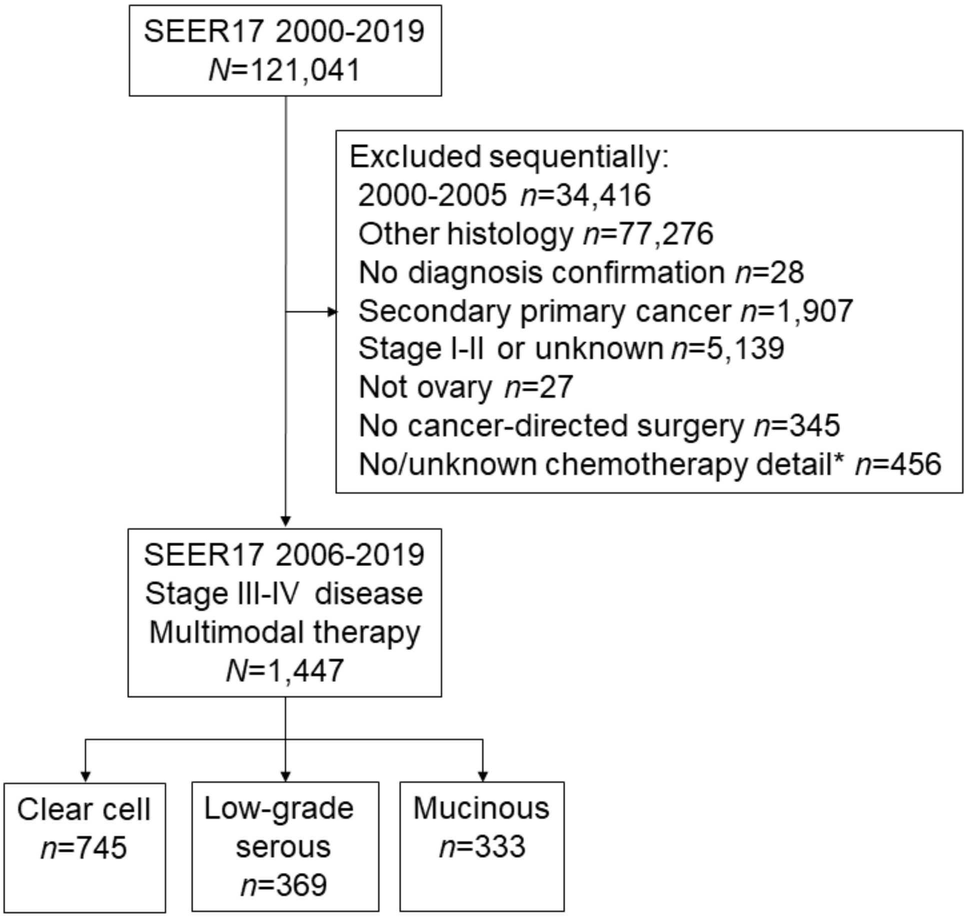

Multimodal therapy refers to cancer-directed surgery and systemic chemotherapy. * including unknown sequence of chemotherapy and cancer-directed surgery and intraperitoneal chemotherapy cases.

© 2023 Matsuo K et al. *JAMA Network Open.*

eFigure 4. Overall survival (SEER cohort).

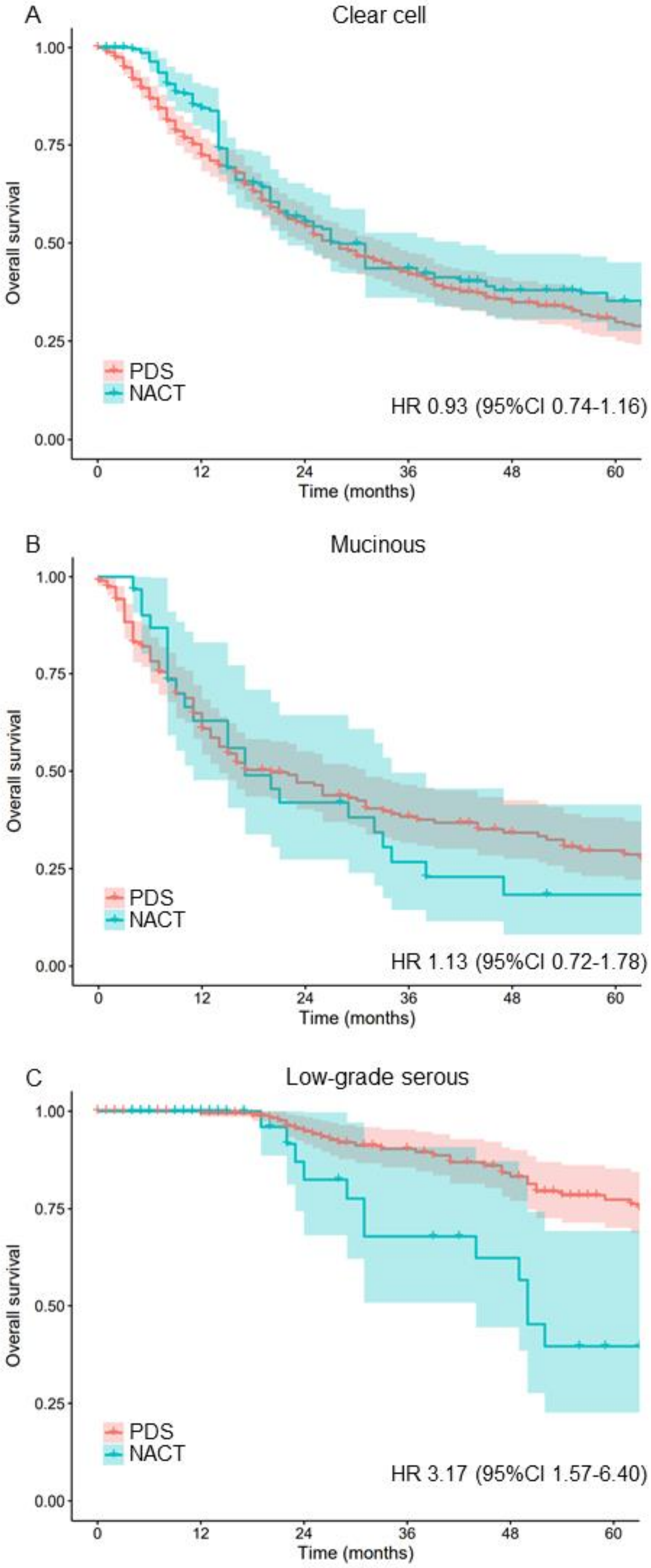

Overall survival based on exposure (NACT vs PDS) are shown for (A) clear cell, (B) mucinous, and (C) low-grade serous carcinomas. The X-axis is truncated at 60 months. Color band widths indicate 95%CI. Abbreviations: HR, hazard ratio; CI, confidence interval; NACT, neoadjuvant chemotherapy; and PDS, primary debulking surgery.

© 2023 Matsuo K et al. *JAMA Network Open.*

eFigure 5. Study selection scheme of the systematic literature search.

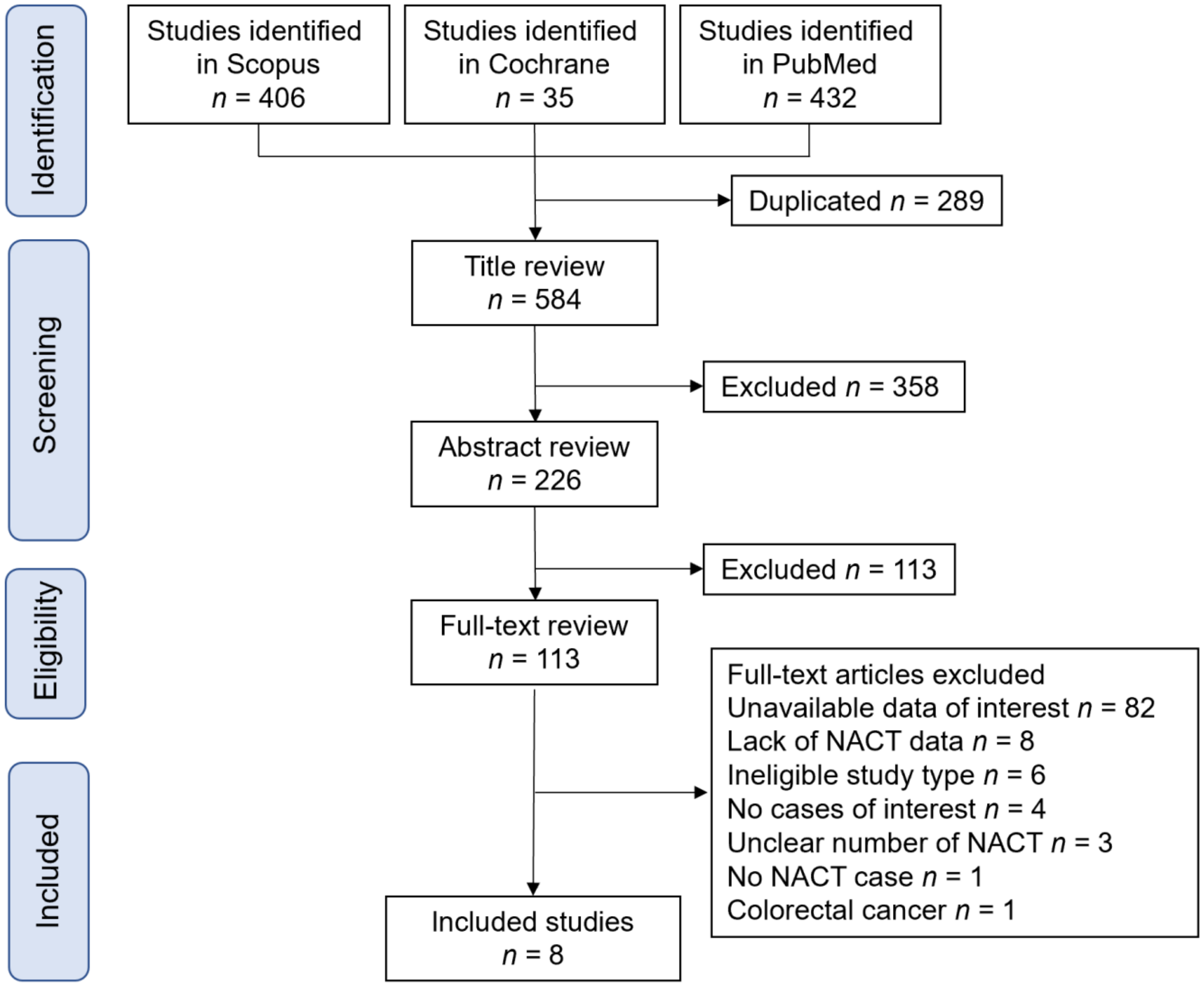

NACT, neoadjuvant chemotherapy.

© 2023 Matsuo K et al. *JAMA Network Open.*

eFigure 6. Meta-analysis (exploratory).

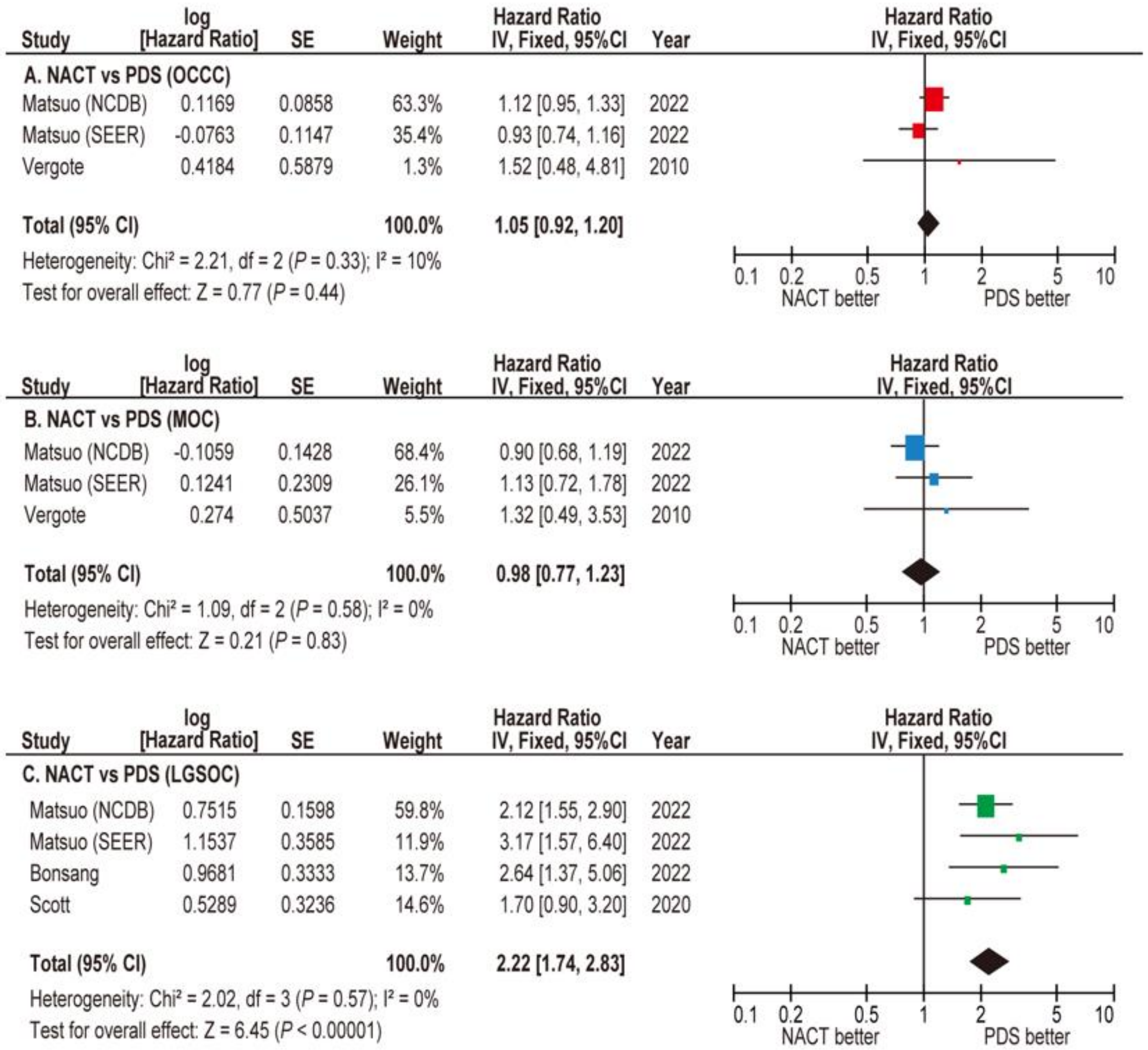

Both main cohort and second cohort of the current study were included. Pooled hazard ratios were calculated using RevMan version 5.4.1 (Cochrane Collaboration, Copenhagen, Denmark). Results of a meta-analysis for the effect of NACT on overall survival in patients with (A) clear cell, (B) mucinous, and (C) low-grade serous carcinomas are shown. A forest plot from a fixed effects meta-analysis of studies for overall survival are ordered within stratum by year of publication and relative weight (%) of studies. Heterogeneity was low among the studies in panel A (I^2=10%), and there was no heterogeneity among the studies in panels B and C (I^2=0%). NCDB, National Cancer Database; SEER, Surveillance Epidemiology and End Results Program; NACT, neoadjuvant chemotherapy; PDS, primary debulking surgery; OCCC, ovarian clear cell carcinoma; MOC, mucinous ovarian carcinoma; LGSOC, low-grade serous ovarian cancer; IV, inverse variable; CI, confidence interval.

© 2023 Matsuo K et al. *JAMA Network Open.*

eFigure 7. Meta-analysis for stage III disease (exploratory).

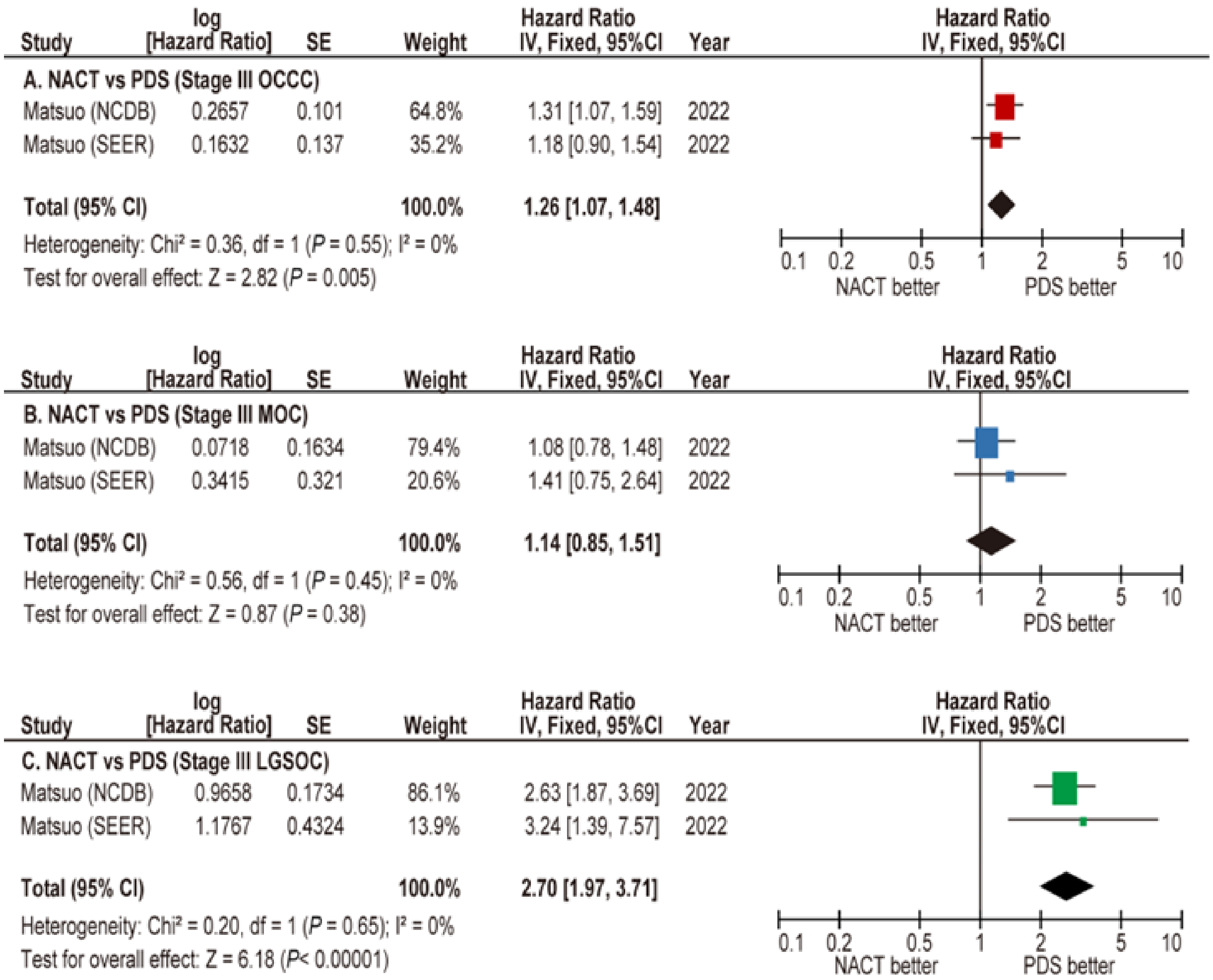

Both main cohort and second cohort of the current study were included. Results of stage III disease for (A) clear cell, (B) mucinous, and (C) low-grade serous carcinomas are shown. *NCDB*, National Cancer Database; *SEER*, Surveillance Epidemiology and End Results program; *NACT*, neoadjuvant chemotherapy; *PDS*, primary debulking surgery; *OCCC*, ovarian clear cell carcinoma; MOC, mucinous ovarian carcinoma; *LGSOC*, low-grade serous ovarian cancer; *IV,* inverse variable; *CI,* confidence interval.

© 2023 Matsuo K et al. *JAMA Network Open.*

eFigure 8. Meta-analysis for stage IV disease (exploratory).

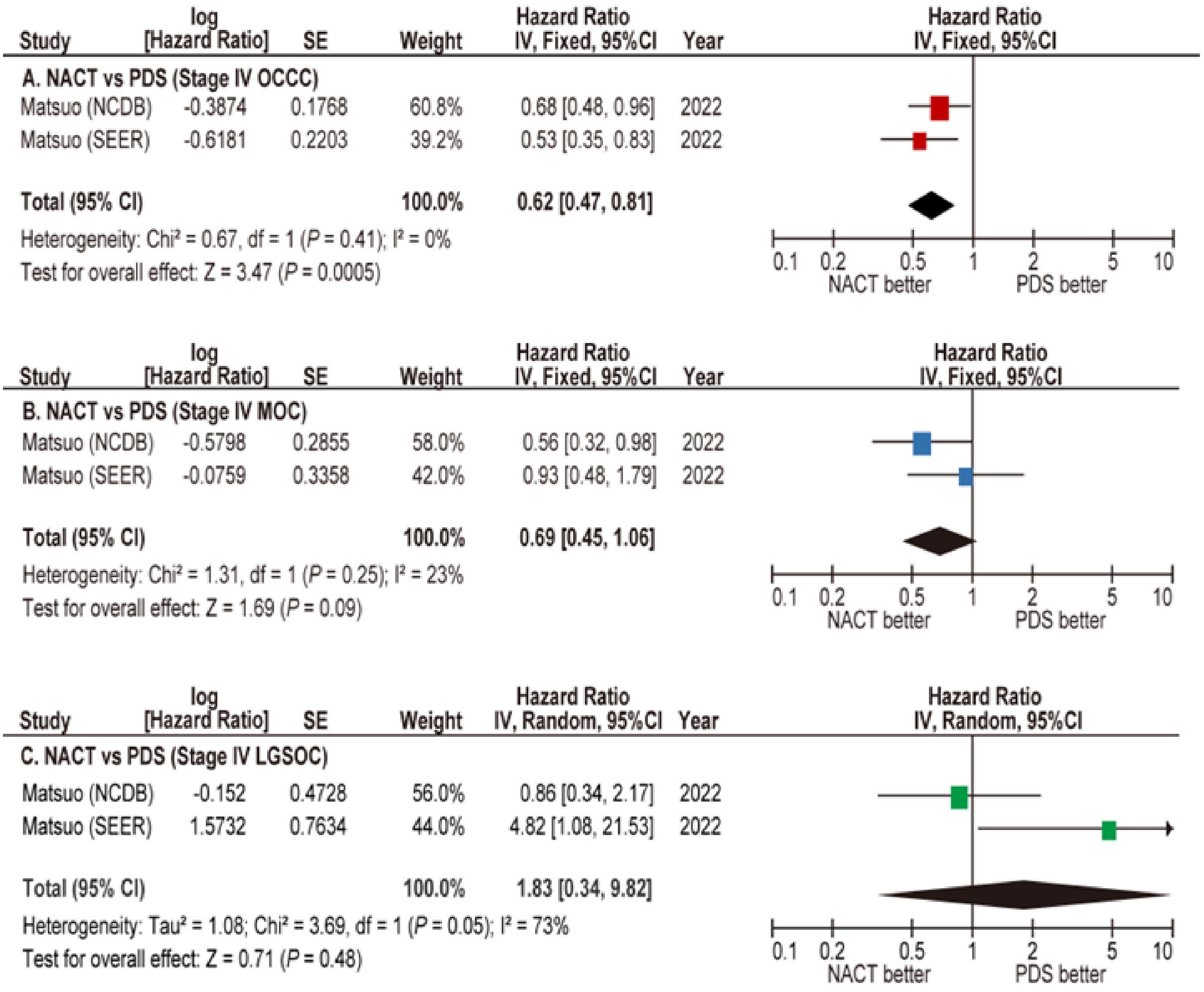

Both main cohort and second cohort of the current study were included. Results of stage IV disease for (A) clear cell, (B) mucinous, and (C) low-grade serous carcinomas are shown. *NCDB*, National Cancer Database; *SEER*, Surveillance Epidemiology and End Results program; *NACT*, neoadjuvant chemotherapy; *PDS*, primary debulking surgery; *OCCC*, ovarian clear cell carcinoma; *MOC*, mucinous ovarian carcinoma; *LGSOC*, low-grade serous ovarian cancer; *IV,* inverse variable; *CI,* confidence interval.

© 2023 Matsuo K et al. *JAMA Network Open.*
