# Supplementary material for: Uptake and Outcomes of Neoadjuvant Chemotherapy Among US Patients With Less Common Epithelial Ovarian Carcinomas
Source: JAMA Netw Open. 2023 Jun 16;6(6):e2318602. doi: 10.1001/jamanetworkopen.2023.18602 (PMC10276312; doi:10.1001/jamanetworkopen.2023.18602)
Supplement: Supplement 2. — Data Sharing Statement [file jamanetwopen-e2318602-s002.pdf]

Data Sharing Statement

Matsuo. Uptake and Outcomes of Neoadjuvant Chemotherapy Among US Patients With Less Common Epithelial Ovarian Carcinomas. *JAMA Netw Open*. Published June 16, 2023. doi:10.1001/jamanetworkopen.2023.18602

Data
Data available: No

Additional Information
Explanation for why data not available: Data Sharing: The data on which this study is based are publicly available upon request at the National Cancer Database (https://www.facs.org/quality-programs/cancer-programs/national-cancer-database/) and the National Cancer Institute's Surveillance, Epidemiology, and End Result Program (https://seer.cancer.gov/).
